# Safe Nanoparticles: Are We There Yet?

**DOI:** 10.3390/ijms22010385

**Published:** 2020-12-31

**Authors:** Wided Najahi-Missaoui, Robert D. Arnold, Brian S. Cummings

**Affiliations:** 1 Department of Pharmaceutical and Biomedical Sciences, College of Pharmacy, University of Georgia, Athens, GA 30602, USA; briansc@uga.edu; 2 Department of Drug Discovery & Development, Harrison School of Pharmacy, Auburn University, Auburn, AL 36849, USA; rda0007@auburn.edu; 3 Interdisciplinary Toxicology Program, University of Georgia, Athens, GA 30602, USA

**Keywords:** nanoparticles, nanotechnology, toxicology, cellular interactions, drug delivery, safe nanoparticles

## Abstract

The field of nanotechnology has grown over the last two decades and made the transition from the benchtop to applied technologies. Nanoscale-sized particles, or nanoparticles, have emerged as promising tools with broad applications in drug delivery, diagnostics, cosmetics and several other biological and non-biological areas. These advances lead to questions about nanoparticle safety. Despite considerable efforts to understand the toxicity and safety of these nanoparticles, many of these questions are not yet fully answered. Nevertheless, these efforts have identified several approaches to minimize and prevent nanoparticle toxicity to promote safer nanotechnology. This review summarizes our current knowledge on nanoparticles, their toxic effects, their interactions with mammalian cells and finally current approaches to minimizing their toxicity.

## 1. Introduction: Nanoparticles and Nanotechnology 

**Figure 1:**
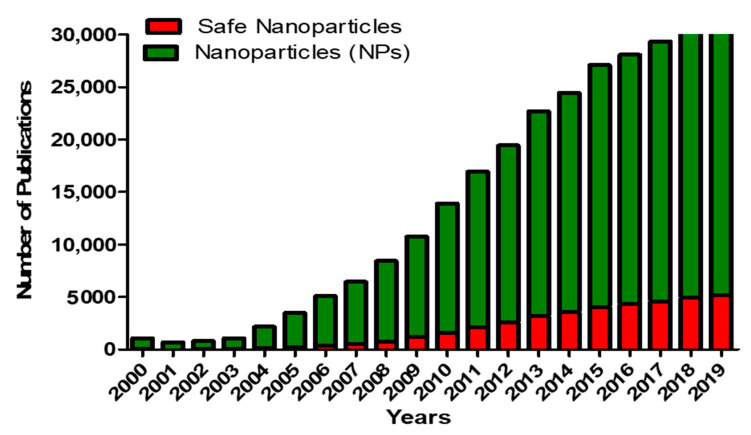
The number of scientific papers published in the last two decades. Papers were identified in Pubmed database from the year 2000 until 2019 using the key words nanoparticles (green) and safe nanoparticles, toxicity of NPs (nanoparticles), risks of NPs (red).

The field of nanotechnology has advanced exponentially in the last decade and many products containing nanoparticles are now used in various applications such as in food science, cosmetics and pharmaceuticals [1]. Nanoparticles (NPs) are defined as particles with one dimension ranging between 1 and 100 nm. NPs exhibit different properties depending on their size and surface functionalities [2]. The small size and large surface area account for the extensive use of NPs in various areas such as cosmetics, electronics and both diagnostic and therapeutic medical applications [3]. The exponential growth and increasing interest in nanotechnology have been enhanced by the ability to image nanomaterials using techniques with atomic resolution capabilities such as scanning tunneling microscopy, scanning transmission electron microscopy and tandem electron microscopy [4,5,6]. Along with the application of NPs, there has been a growth in scientific publications, as shown in Figure 1. 

The exponential growth of nanotechnology has led to studies focusing on the associated risks of NPs and nanotechnology in general. However, and despite our increased exposure to NPs, information regarding NP safety is lagging behind as compared to the research on the application of NPs (Figure 1) [3]. 

NPs are used as pharmaceutical drug carriers with applications in both diagnostics and therapy. These NPs, including polymeric NPs, nanoemulsions, liposomes and solid NPs, are suggested to have potential clinical applications. Their clinical applicability depends on different parameters such as their physical and chemical properties, drug loading efficiency, drug release and most importantly low or no toxicity of the carrier itself [7]. 

Despite the potential for clinical application, some studies have suggested that NPs can be toxic. These studies have demonstrated the ability of NPs to accumulate in cells and induce organ-specific toxicity. These studies, combined with the ever-increasing human exposure, demonstrate an urgent need for the design of safe NPs and the development of strict guidelines for their development with regards to toxicity testing.

This review briefly introduces the reader to the major types of NPs and their various biomedical applications. The reader is referred to recent publications for a more complete review of the types and application of NPs [3]. This review then summarizes the current understanding of NPs’ cellular interactions and their current toxicological status. The last section of this review identifies key properties of NPs that correspond to decreased toxicity and presents approaches toward producing safer NPs to further facilitate the use of nanotechnology.

## 2. Nanoparticles and Their Applications

### 2.1. Polymer-Based Nanoparticles

Polymeric nanoparticles (PNP) are used widely as drug carriers for controlled and sustained release. The encapsulated entity can be attached to the surface of a nanosphere or nanocapsule, or incorporated into a matrix or shell of the polymer [8]. Two commonly used polymers are poly lactic glycolic acid (PLGA) and chitosan, which have both been FDA approved for clinical use, partially due to the fact that they are biocompatible and biodegradable [9,10,11].

### 2.2. Solid Nanoparticles: Iron Oxide, Gold and Silver Nanoparticles

Solid NPs include iron oxide, gold, silver and other metal-based NPs. Iron oxide NPs are produced by conjugating an organic core of magnetite (Fe_3_O_4_) or maghemite (Fe_2_O_3_) to a biocompatible polymer. Iron oxide NPs have attracted great interest in the last decade, primarily because of their superparamagnetic properties [12,13]. Iron oxide NPs are used in biosensors in combination with magnetic resonance imaging (MRI), targeted drug and gene delivery and magnetic fluid hyperthermia [14]. In addition, iron oxide NPs have been used in various diagnostic and imaging techniques because of their unique optical properties that enable them to act as biosensors in living cells [15,16]. 

Depending on their size, shape and surface properties, gold NPs have been suggested for use in cancer diagnosis, therapy and radiotherapy [17,18]. Gold NPs can even be used to develop theranostic systems where diagnosis, imaging and therapeutics can be combined for improved therapy [19,20]. Gold NPs were shown to have reduced toxicity as compared to other solid NPs; however, a full understanding of their toxicity profile is still lacking [21]. 

Silver NPs are another class of solid NPs that have received some attention. This includes the use of silver NPs as biosensors because of their optical properties and ability to absorb and scatter light. Silver NPs are heavily used in electronic devices, textiles, wound dressings, antimicrobial coatings and biomedical devices [22,23].

### 2.3. Carbon-Based Nanoparticles

Carbon-based NPs are being increasingly used in various biomedical applications such as drug delivery, gene therapy and imaging. Carbon nanotubes (CNTs), a major class of these NPs, include single-walled (SWCNTs) and multi-walled (MWCNTs) types. CNTs’ unique physiochemical properties make them strong candidates for various applications in biomedical fields, such as drug and gene delivery, biosensors and tissue engineering applications [24,25,26,27]. They also display high stability and have specific surface chemistry that allows for increased drug loading capacity. However, the safety of CNTs is still questionable as they have been shown to be toxic to healthy tissues following chronic exposure [27,28,29,30]. 

### 2.4. Lipid-Based Nanoparticles 

Liposomes are the classic example of vesicular lipid-based NPs. Liposomes consist of a phospholipid and cholesterol bilayer that entraps an aqueous core, as first described in 1965 [31]. Liposomes are one of the most commonly used drug delivery systems, with several currently FDA-approved formulations in use and many more in clinical or preclinical trials [32,33]. Liposomes have been used for drug, nutraceutical and biologic-based delivery and can exhibit high encapsulation efficiency and extended circulation time. These properties enable liposomes to accumulate at specific sites of disease, such as tumors, where the endothelial layer is discontinuous, a functional lymphatic system is lacking and liposomes accumulate passively via the enhanced permeability and retention effect (EPR) [34,35]. Early conventional liposomes had limitations such as short half-life and rapid systemic clearance following their opsonization and clearance by the reticuloendothelial system (RES). However, the conjugation of polymers such as polyethylene glycol (PEG) or PEGylation of liposomes, and the use of saturated high-phase transition lipids, resulted in the generation of sterically stabilized liposomes or “Stealth^TM^ liposomes” (SL), with prolonged half-life and increased stability [36,37,38]. The long systemic circulation time of Stealth^TM^ liposomes allows for altered biodistribution and greater accumulation in solid tumors, compared to conventional formulations [39,40]. Among their advantages, liposomes and other lipid-based NPs have been shown to have the least toxic effects for *in vivo* applications [7].

### 2.5. Nanoemulsions

Nanoemulsions (NE) have been commonly used in the delivery of vaccines and anti-cancer agents. NEs are colloidal dispersions that can be used as drug carriers of molecules with limited water solubility [41]. NEs are usually composed of either oil nanodroplets dispersed in water or water nanodroplets dispersed in oil. Surfactants are typically included to enhance their stability. However, a key limitation of these carriers is that they are thermodynamically unstable if their size exceeds 500 nm. In contrast, NEs in the range of 20 to 200 nm are more thermodynamically stable [42,43]. 

NEs are suggested to be suitable for various administration routes including parenteral, transdermal and ocular, mostly due to their ability to protect encapsulated drugs from enzymatic degradation and hydrolysis [44]. NEs, like many of the NPs discussed above, can be modified by conjugating them to different ligands to target macromolecules selectively expressed in specific diseases such as cancer. For example, ligand-conjugated NEs, which can target receptors whose expression is increased in certain cancers, have been shown to have enhanced uptake into cancer cells, facilitating reduction in tumor growth [45].

### 2.6. Nanoparticles in Biomedical Applications 

Various NPs including liposomes, iron oxide NPs and polymeric NPs are being tested in clinical and preclinical studies for drug and gene delivery with the goal to improve their site-specific delivery [46,47,48]. Many drugs with therapeutic efficacy unfortunately have poor aqueous solubility and their encapsulation in NPs can improve their stability by minimizing precipitation and reducing the need for toxic cosolvents. NPs can also alter rates of drug metabolism and clearance and therefore improve drug efficacy [49,50,51]. For example, the FDA approved doxorubicin liposomes Doxil^®^ (Centocor Ortho Biotech, Horsham, PA, USA) for the treatment of metastatic breast cancer and Kaposi’s sarcoma. These nanoparticles have significantly increased efficacy compared to free drug [52,53]. 

Conventional or first generation NPs do not display specific cell targeting, however, targeting moieties can be conjugated to NPs to enhance delivery to specific cells. As mentioned above, NPs can be actively targeted to different disease sites by conjugating them to specific ligands capable of recognizing and binding to membrane proteins known to be overexpressed in various pathologies, such as in cancer [54]. 

NPs are also used as gene delivery systems and have shown efficacy in replacing specific target diseased genes involved in cancer, certain viral infections and other genetic disorders. Unfortunately, cationic NPs can cause immunological responses that limit their use [55,56,57]. Additionally, NPs have been used in cellular imaging to detect cellular changes *in vitro* and *in vivo* [58]. NPs can be conjugated with moieties such as antibodies and other ligands to increase their targeting efficiency [2]. 

Despite their promising efficacy as drug or gene carriers, there are fewer NPs in clinical use than one would predict based on the wide preclinical studies. This is mainly because of potential toxicity by mechanisms that are not fully understood, which is especially true for NPs being administered chronically [59].

## 3. Mechanisms of Nanoparticles Toxicity

**Figure 2:**
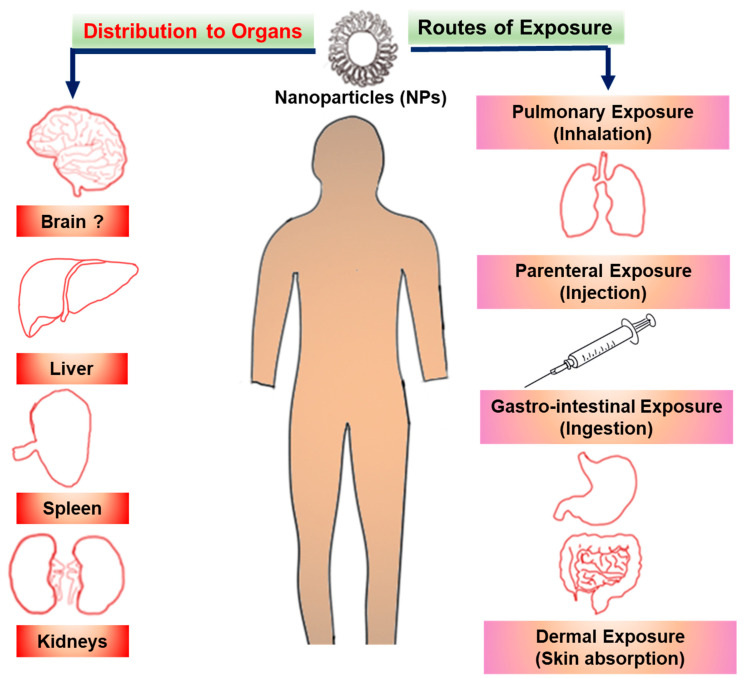
Routes of exposure to NPs and their distribution to organs in the human body. Exposure to NPs may be through lungs, injection, ingestion or skin absorption. Distribution organs include liver, spleen and kidney. The brain is suggested as a potential target for NP distribution, however direct evidence is still lacking.

Like any drug or chemical, the toxicity of NPs is dependent on the route of administration and their exposure [3]. Exposure to NPs may occur through ingestion, injection, inhalation and skin contact (Figure 2). The organ-specific toxicity following exposure to NPs depends on the route of administration and their systemic distribution. Some exposures are unintentional, such as in pulmonary inhalation of NPs in the environment or manufacturing places. Exposure through the lungs can result in inflammatory reactions, fibrosis and necrosis of lung tissues [60,61]. Other exposures are intentional, such as application of skin products or drug administration via ingestion or injection. 

Once they reach the blood circulation, NPs can be distributed and can accumulate in different organs such as the liver, spleen, lungs and kidneys. Some studies suggest that NPs may also accumulate in the brain if they are small enough (<10 nm) and/or the blood brain barrier is not intact. However, there is still a lack of evidence of nanoparticle transcytosis [62,63]. A common property of most of these organs is their leaky blood vessels, which is also true for tumors. This leaky vasculature enhances the accumulation of NPs because of the EPR effect [64,65]. Accumulation of NPs in specific organs also depends on their physical and chemical properties. In addition, aggregation of NPs may affect their biodistribution and organ deposition. Recent studies using different animal models showed that NPs accumulate in different organs, interact with cellular macromolecules and cause oxidative stress [66,67]. NP distribution depends on their surface area to size ratio, which also mediates their propensity to accumulate in different tissues and organs [68,69]. 

Much of what we know about NPs’ toxicity and distribution comes from studies performed using short-term exposures. As such, there are fewer studies assessing the toxic effects of NPs following chronic exposure. In addition, the pharmacokinetics of NPs as drug delivery systems are not well studied [65,70]. Most studies focus only on the pharmacokinetics of the encapsulated drugs, as opposed to the drug carriers themselves. This creates a strong need for more studies on the toxicokinetics of NPs as carriers in blood and different organs [59]**.**

**Figure 3:**
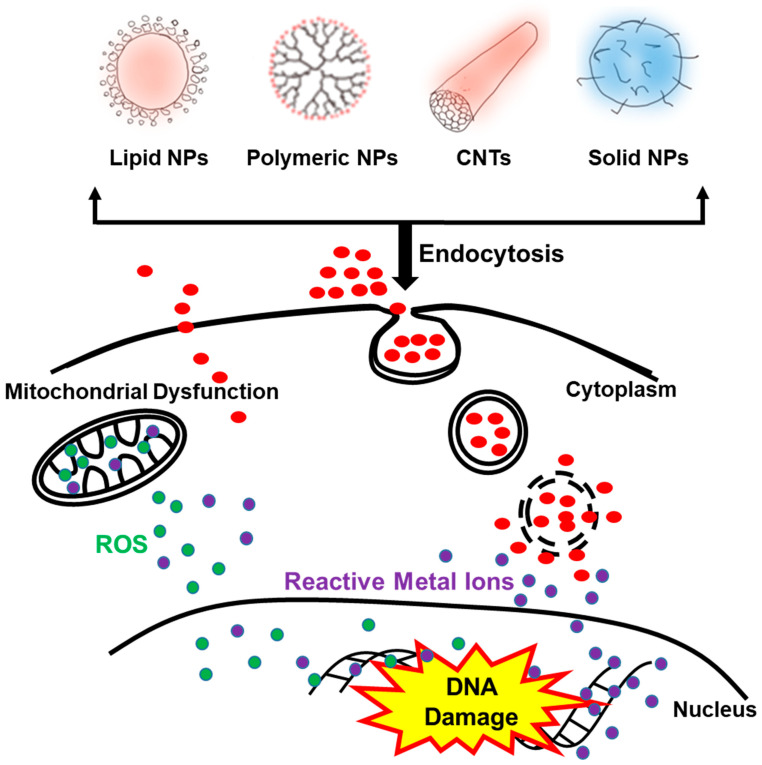
Cell–nanoparticle interactions and mechanism of NP toxicity. Following cellular uptake of NPs, NPs go through lysosomal pathways and result into release of reactive oxygen species and/or reactive metal ions causing mitochondrial dysfunction and DNA damage.

The mechanisms mediating the toxicity of NPs in their target organs include the generation of reactive oxygen species (ROS), DNA damage, modification of protein structures and functions and disruption of membrane integrity (Figure 3). NP properties that appear to facilitate these mechanisms include large surface areas that facilitate molecular interaction in the target sites. 

After acute systemic exposure, solid NPs such as metal-containing or metal oxide NPs have been shown to cause oxidative stress, inflammation and DNA damage [71,72]. These NPs have been shown to induce oxidative stress in the liver, spleen and kidneys and inhibit the effects of antioxidants [73,74,75]. NP-induced ROS result, in part, from mitochondrial dysfunction and activation of stress-related cell signaling pathways, as well as DNA damage, that results in cell cycle arrest and apoptosis (Figure 3) [74,76]**.** Other studies have suggested that NP-induced oxidative stress may result from modification of catalase functions and protein oxidation. Some NPs, like titanium dioxide, have been shown to induce the release of nitric acid and glutamic acid in the brains of mice [77,78]. Titanium dioxide NPs have also been suggested to activate inflammatory signaling pathways via c-Src, p38 MAP kinase and NF-κB pathways, and to increase the release of inflammatory cytokines from macrophages after their accumulation in macrophage-rich organs, such as the liver and spleen [79,80,81,82]. 

Some NPs have been suggested to induce epigenetic modifications, such as modifications in the methylation status of DNA and post-translational changes of histones, in addition to chromatin remodeling and RNA methylation. However, more studies are needed to verify and explain the mechanisms and the biological effects of these epigenetic modifications [83,84,85,86]. 

## 4. The Effect of NP Properties on Their Toxicity 

**Figure 4:**
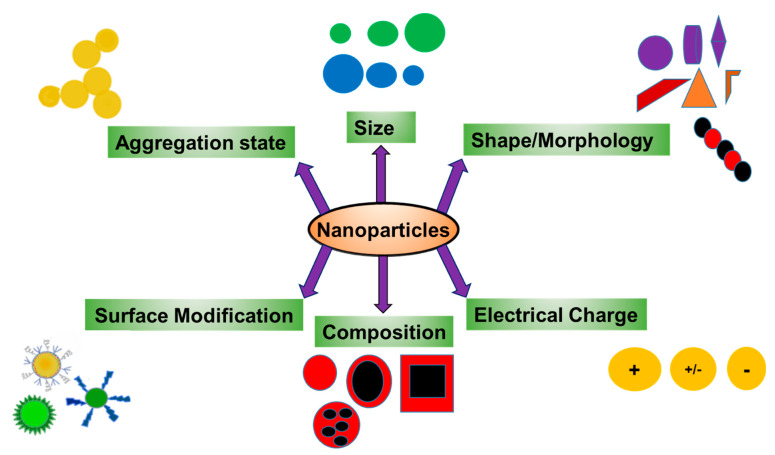
Physicochemical properties of nanoparticles that dictate their toxicities. Size, surface modification, surface charge, composition, shape and aggregation state of NPs are key factors in dictating NPs distribution in different organ systems following their exposure.

The toxicity of NPs is dependent on their biophysical properties including size, surface area, surface charge and aggregation state (Figure 4) [59]. These properties have been shown to affect distribution and deposition of NPs in different organ systems and alter their molecular interactions with various proteins and other macromolecules [87]. 

NPs’ size plays an important role in their entry route, cellular uptake and overall toxicity. Some studies suggest a direct correlation between the size of NPs and their distribution and the level of ROS generation in the kidneys [88,89]. This size-dependent toxicity has also been shown in the liver. Silver NPs with a size of 10 nm exhibited higher tissue distribution and caused more serious toxic effects in the liver compared to larger NPs (40 and 100 nm) [90,91]. 

Along with their size, the shape of NPs can also affect their distribution, deposition and clearance. Long fibrous particles, such as single-walled nanotubes, are difficult for the body to clear and therefore exhibit high organ deposition [92].

The surface chemistry of NPs can affect their pharmacokinetics. Charged NPs tend to exhibit higher accumulation in target organs than uncharged NPs. The ionic forms of zinc oxide NPs were shown to accumulate more in organs like liver, lungs and kidneys following oral or intravenous administration compared to uncharged NPs [93]. 

The conjugation of polysaccharides was also shown to enhance the accumulation of NPs in the brain, liver and spleen, which correlated to their toxicity in these organs [94]. On the other hand, modification of NP surface properties using different coating materials can be used to reduce their potential toxicity. Polyethylene glycol (PEG) has been shown to reduce the toxicity of NPs by altering their interactions with proteins. However, it should be noted that increasing the amount of PEGylation may alter NPs’ cellular uptake and their efficacy as drug delivery systems [95,96]. 

## 5. Nanoparticle–Cell Interactions: Uptake of Nanoparticles into Cells 

The primary determinate of NP efficacy or toxicity is their ability to interact with the cell. Just as with target organ toxicity, the size of NPs plays a major role in their cellular interaction and also determines their systemic circulation half-life and their biodistribution [97]. NPs cellular uptake differs from that of small molecules and understanding these differences is critical to understanding and hopefully limiting NP cellular toxicity [98]. Factors influencing NP–cell interactions and uptake are most likely related to the physicochemical properties of NPs and the biophysical properties of cell membranes. It is important to know the mechanism of NP cellular uptake and the relationship between their physical–chemical properties and their toxicity. This can dictate the fate of NPs and help understand their potential toxic effects and therefore help design efficient and safe NPs [99].

Endocytosis appears to be the major pathway of cellular uptake of NPs (Figure 3). Once taken up by cells, NPs have been suggested to remain in the intracellular compartments for weeks or even months [100,101]. Despite this knowledge, the contribution of endocytosis to the cellular toxicity of NPs is not well understood. Endocytosis of NPs is energy-dependent and usually facilitated by their binding to membrane receptors such as integrins or growth factor receptors. The extent of endocytosis depends on the size of NPs and since smaller NPs are more likely to be internalized inside cells than larger NPs, they are more likely to cause cellular toxicity. In addition to size, surface chemistry, concentration, time of exposure and clearance of NPs can all modulate the extent of cellular endocytosis and toxicity of NPs [98,102]. Endocytosis of NPs can happen via different mechanisms. These include phagocytosis, pinocytosis, clathrin-dependent endocytosis, caveolae-dependent endocytosis and clathrin/caveolae independent endocytosis [103,104,105,106]. Studies have shown that the shape of NPs can affect their systemic circulation time and therefore delay their cellular uptake. For example, Champion et al. have shown that when compared to spherical polymeric NPs, non-spherical NPs exhibit less phagocytotic uptake by macrophages and therefore longer circulation time [107]. NPs can also be internalized through membrane translocation or simple diffusion, which are energy-independent mechanisms depending on the concentration gradient of NPs in addition to their size and lipophilicity [108]. Cellular interactions and uptake of NPs can be affected by the extent of NP modification. Gold nanoparticles with amino acids such as aspartate, glycine, leucine, lysine and serine conjugated to their surface were shown to adsorb serum albumin. Albumin adsorption decreased intracellular uptake of these NPs and improved their biocompatibility compared to gold NPs with no conjugated amino acids [109].

Within cells, NPs have been found free in the cytoplasm or enclosed by a membrane [110,111] (Figure 3). A study by Verma et al. showed the role of surface coatings on the cellular uptake of gold NPs. Hydrophilic gold NPs were enclosed by a membrane during the cellular uptake, while partially hydrophobic NPs were shown without surrounding membranes in the cells [112]. Once inside the cells, some NPs undergo the endosomal/lysosomal pathways and the different cytoplasmic networks. Despite this knowledge, the mechanisms dictating the distribution of different NPs to intracellular compartments and the effect of this distribution on NP toxicity are not fully understood. However, it is believed that the extent of cellular retention may be major factor in NP toxicity. Therefore, limiting how long NPs reside intracellularly while maintaining the efficacy of encapsulated drugs is a key factor in reducing their toxicity [74,113]. 

## 6. Acute Toxic Effects of Nanoparticles after Cellular Uptake

Much of our knowledge about the toxicity of NPs and their cellular uptake comes from diagnostic and therapeutic studies designed to treat human diseases. As mentioned above, these studies show that the toxic effects of NPs are dependent on their size, shape, their chemical composition and their extent of agglomeration (Figure 4) [114,115,116]. These studies also suggest that the toxic responses of NPs should be assessed by normalizing them with their uptake and their state of agglomeration, as opposed to their initial dose. Crystallinity and composition should also be considered when assessing NP toxicity, both of which should be correlated to particle size distribution [117].

Like all drugs, the dissolution of NPs can affect acute toxicity. Unlike polymeric NPs or lipid-based NPs, inorganic NPs such as metal oxide NPs including zinc, iron and silver oxide NPs, are believed to dissolve after exposure, and it is believed that the release of free ions associated with these inorganic NPs contributes to their toxicity (Figure 3). Studies show that insoluble nanoparticles of ceria (cerium oxide), titania (titanium dioxide) and zirconia (zirconium dioxide) showed no measurable toxic response when used in human mesothelioma cells following six days of exposure at concentrations up to 30 ppm (μg/mL). In contrast, soluble nanoparticles of iron oxide and zinc oxide were toxic at similar concentrations after three days of exposure [117]. This study is supported by several others suggesting that the solubility of inorganic NPs is a key property in assessing their acute toxicity [118,119]. 

Following their cellular uptake, NPs are observed in lysosomes where the acidic pH of 5.5 assists in their degradation and dissolution to release potentially toxic heavy metal ions. These ions have been suggested to damage the cell by various mechanisms such as ROS formation and inactivation of enzymes (Figure 3) [118,120]. This hypothesis is supported by numerous studies showing that NPs increase the number of free radicals inside cells in a dose-dependent manner. The generation of free radicals can result from either a reduction or a catalytic process. This poses a great concern as catalytically active NPs can generate radicals repeatedly, until they are degraded or removed from the body. Therefore, catalytically active NPs pose a significant concern when used chronically, a very under-studied area as many studies focus typically on acute toxicity [121,122]. 

## 7. Nanoparticle Toxicity Following Chronic Exposure

Acute toxicity assessment of NPs is not sufficient to evaluate their safety for many reasons. First of all, exposure to NPs is a continuous daily process, such as exposure of workers in the manufacturing sector, or exposure through daily applied cosmetics. Secondly, the dissolution or degradation of NPs may take a significant amount of time, possibly much longer than the elimination of the therapeutic they are carrying. Further, the dissolution or degradation products of NPs may themselves be toxic. Finally, the biodistribution and accumulation of NPs may change over time [123]. This creates the need for further studies regarding the chronic exposure outcomes of NPs. These should include studies assessing the chronic use of NPs in humans both in clinical and industry settings, as well as those assessing bioaccumulation in the environment. Chronic exposure conditions need to be studied differently from acute exposure since they could involve several steps that cannot be simulated in a single-step acute toxicological exposure [123,124]. 

This is supported by several studies examining the possible chronic toxicity of NPs. For example, studies led by Poland and Kane showed that carbon nanotubes administered into mice via the abdominal cavity caused asbestos-like pathogenicity [125,126]. Another study showed later that long-term exposure to multi-walled carbon nanotubes can promote breast cancer metastasis [127]. Chronic exposure to nanoparticles can cause genotoxicity, carcinogenesis and embryotoxicity (developmental toxicity) [123]. Chronic oral administration of aluminum oxide nanoparticles and zinc oxide nanoparticles into rats daily for 75 days caused hepato-renal toxicities and suppression of the hepatic expression of mtTFA and PGC-1α proteins [128]. Chronic exposure (21 days) of the planktonic crustacean Daphnia magna to gold NPs caused mortality of parental females, impaired development and decreased reproductive fitness manifested by reduction of the total offspring and aborted eggs [129]. Li et al. (2018) showed impairment of the transcription of key genes involved in DNA damage/repair, antioxidation and apoptosis, such as p53, PDRP, SOD, CAT and GST, in the marine mollusk Mytilus galloprovincialis following chronic exposure to zinc oxide NPs [130]. After a 12 week inhalation experiment in rats exposed to titanium dioxide NPs, Oberdorster and his group reported inflammation, lung injury and impairment of alveolar macrophage function [131]. 

Chronic studies of NP toxicity should endeavor to cover all of the toxicology observations (hematology, analysis of organs and tissues, genetic analysis). Further, such studies should use proper power analysis to ensure that an efficient number of animals are used to enhance the rigor of the data. In some cases, such as those assessing the carcinogenicity of NPs, studies may need to be conducted over the life-time of the animals studied (typically 2 years for rodents) [123]. In addition, as with all *in vivo* chronic toxicity studies, route of exposure, dose, frequency and duration of exposure, animal age and sex need to be considered, along with understanding the physicochemical properties of the NPs involved, including their composition, size, shape, charge, aggregation status and degradation [88,132].

## 8. How Safe Are Nanoparticles and How Can We Make Safe Nanoparticles? 

The above studies demonstrate the growing concern about the toxicity of NPs and stresses the need to consider NP toxicity during their initial design, whether this be for industrial or clinical use. One approach being used in the design of safer NPs can be highlighted by recent developments in structured nanoemulsions and solid lipid nanoparticles. These nanoparticles are being formulated using food grade ingredients that have been generally recognized as safe (GRAS) by the FDA, including lipids, proteins, polysaccharides and surfactants [133]. Studies have also shown that many toxic effects of NPs are associated with solid or metal-containing NPs. Thus, efforts are being made to limit their use. Other approaches are discussed below. 

### 8.1. Defining the Risk of Nanoparticles 

A practical approach to achieve safer nanotechnology would be to classify potential risks of different NPs. Classification would be based on current toxicological assays assessing acute toxic effects and classification based on potential long-term effects. This approach has the benefit that current toxicological assays used for chemicals can also be used for degradable NPs that have short residence times in the human body or the environment. These assays assess toxic responses of the NPs as well as their degradation products. 

Unfortunately, many toxicological assays cannot accurately predict toxic effects after chronic exposure and assays for NPs with long residence times are needed to more accurately predict potentially adverse long-term effects. This approach is hampered by the fact that the residence times for many NPs are not known, especially in humans. This necessitates the need to consider the circulation of NPs in the body or environment during their initial testing [59,134]. 

This also necessitates the need to assess the risk of persistent NPs differently than those for degradable NPs. Persistent NPs would be those that stay in the mammalian body or in the environment for a prolonged time. Unfortunately, there is a gap in the knowledge with regards to the chronic effect of persistent NPs. As such, some have proposed that persistent NPs must be considered as “potentially hazardous” and should be handled with special care [135].

Degradable NPs are those that are either degraded or metabolized fairly quickly in the body. As such they do not reside in and have brief interaction with cells. Therefore, they have shorter times of exposure and their degradation or metabolic products can be evaluated with currently used standard methods, similar to that used for chemicals. 

Whether or not NPs are persistent or degradable, it is crucial to emphasize that the pharmacokinetic properties of NPs such as their distribution and elimination should be taken into account [136]. Unfortunately, many approaches to determining the pharmacokinetic properties of NPs were designed for single chemical entities. As such, these approaches may not be accurate for NPs. This creates a critical need to develop standard methods to assess NP pharmacokinetics for therapeutic and other purposes, especially after long-term/chronic exposures. 

### 8.2. Approaches to Produce Non-Cytotoxic Nanoparticles 

One promising approach toward decreasing the risk of some NPs, especially lipid-based NPs, is the use of “next-generation lipids” that combine high potency and biodegradable properties. Martin et al. have developed biodegradable lipids by incorporating biocleavable ester functions within the hydrophobic alkyl chains. This class of biodegradable lipids showed rapid elimination from plasma and improved tolerability in preclinical studies with high *in vivo* potency [137]. 

Surface coating strategies are also being suggested as one of the major surface modification strategies to decrease the risk of NPs and design safer nanotechnology. Surface coating refers to any modification, functionalization or stabilization applied to NPs in order to selectively alter their properties. The surface of NPs can be covered with various substances such as polymers in single- or multi-layers that can be either complete or incomplete [138]. This is because the coating material, if chosen correctly, provides biocompatibility and affects the behavior (e.g., colloidal stability) and the fate (e.g., degradation, excretion, accumulation) of NPs following their administration in the complex environment of biological fluids, cells and organisms. 

There are numerous coating materials and coating techniques that are available for NPs. However, the most important criteria for these coating materials is to maintain high colloidal stability from the production steps of NPs, including stability in salt- and protein-containing media, such as buffer solutions or cell culture media, to their *in vitro* testing in biological cells and *in vivo* testing in animal models [138]. Another advantage of this strategy is that surface coating is reversible by non-covalent modification. Since bioavailability and potential toxicological effects of NPs are dependent on their dispersion state, various noncovalent coatings can be used to alter the dispersion state of NPs to alter their toxicity [139]. Coating strategies can be used for various types of NPs including polymeric, lipid-based and inorganic NPs. Examples of coating materials include polyethylene glycol (PEG), polyvinylpyrrolidone (PVP), polyvinyl alcohol (PVA), poly(N–isopropylacrylamide) (PNIPAM), zwitterionic polymers such as poly(carboxybetaine) (PCB), poly(sulfo-betaine) (PSB) and phosphorylcholine-based copolymers and polysaccharides such as dextran and chitosan [138,140,141,142]. As an example, single-walled and multi-walled carbon nanotubes (CNTs) have been used in drug delivery as drug nanocarriers, in tissue engineering, water purification and in sensors [143,144]. CNTs have been also shown to induce inflammation, fibrosis and promote cancer progression as a result of their surface chemistry, length and aggregation state [144,145]. Wang et al. have shown that surface coating of CNTs using a nonionic triblock copolymer, PF108, improved the dispersion state of CNTs and reduced their agglomeration, cellular uptake and pro-fibrogenic effects. The authors assessed the protective effects of PF108 coating against the toxicity of CNTs *in vitro* using bronchial epithelial BEAS-2B cells and phagocytic THP-1 cells and *in vivo* using mice lungs [146]. The decrease in toxicity correlated to a decrease in pro-inflammatory cytokine (IL-1β) production by THP-1 cells and pro-fibrogenic TGF-β1 production by BEAS-2B cells, as compared to non-coated CNTs. *In vivo* studies demonstrated that PF108-coated CNTs reduced their deposition in the lung and protected against pulmonary fibrosis compared to uncoated CNTs. The stability of the PF108 coating on CNTs was maintained even under acidic lysosomal conditions [146]. Another study conducted by Mutlu et al. demonstrated that CNTs coated with PF108 protected against lung toxicity and were cleared from the lungs after 90 days compared to non-coated CNTs, which aggregated and induced granulomatous lung inflammation and fibrosis [147]. These studies suggest that surface coating with pluronic F108 (PF108) can provide protection against particle-induced toxicity and may be an effective strategy for the design of safer NPs.

Doping is a widely used and effective strategy for inorganic NPs. This technique alters the crystal structure of materials through the addition of impurities to improve chemical and physical properties [148,149,150]. Examples of dopants include aluminum, titanium and iron. These dopants, when incorporated evenly into the nanoparticles, have shown the ability to alter the density of reactive chemical entities on the surface of NPs and therefore reduce the binding energy of metal ions to oxygen [151,152]. Doping of NPs can decrease NP dissolution and cause a reduction in toxic ions released, and therefore alter the reactive surfaces, resulting in a decrease in ROS generation [152,153,154]. 

Doping has increased the potential use of inorganic NPs in nanomedicine. Inorganic NPs have been studied extensively; however, they also display significant toxicity to healthy cells and organs, which limits their clinical applications [3,155]. Doping has been shown to improve the antimicrobial potential of silver-based NPs, which, when doped with titanium oxide (TiO_2_), enhanced their antibacterial activities against *Escherichia coli* and *Bacillus subtilis* [156]. 

Flame spray pyrolysis (FSP) is a well-established technique used in NP doping. FSP uses a rapid combustion method, liquid precursor, self-sustaining flame with a high local temperature and large temperature gradient that allow for the synthesis of homogenous crystalline nanoscale materials [157]. Even though ZnO NPs have wide industrial applications, such as in cosmetics (e.g., sunscreens) and electronics [158], ZnO-induced pulmonary inflammations have been reported in humans. This phenomenon is known as “metal fume fever” that takes place when welders are exposed to metal fumes containing high concentrations of ZnO [159]. This suggests that reduction of dissolution of ZnO could possibly decrease these toxic effects [160]. George et al. (2010) synthesized Fe-doped ZnO NPs by FSP and assessed their cytotoxicity *in vitro* using RAW 264.7 and BEAS-2B mammalian cells and found that ZnO dissolution was decreased, which correlated to reduced cytotoxicity [151]. *In vivo* studies showed the reduced toxicity of Fe-doped ZnO nanoparticles in zebrafish embryos and rodent lungs [153]. While doping seems to be promising in reducing toxic effects of NPs, further studies are needed to determine if doping has any interference with the efficacy of encapsulated drugs and therefore their clinical applications. 

Other modifications of surface properties of NPs that have been suggested to reduce their risk include alteration of charge density and hydrophobicity, which is reported to improve the efficacy of some NPs in biomedical applications including targeted drug delivery [161,162,163,164]. Adjustment of surface chemistry properties of NPs can be achieved by covalent binding of functional groups such as anionic, nonionic and cationic groups onto their surface [164,165,166,167,168,169]. Li et al. (2013) synthesized and assessed the toxicity of CNTs functionalized with anionic, nonionic and cationic surface groups *in vitro* and *in vivo*. CNTs with the anionic groups (carboxylate and polyethylene glycol), displayed the lowest pro-fibrogenic effects and uptake in THP-1 and BEAS-2B cells [166]. Cationic CNTs interact with anionic groups on cell membranes, which appears to enhance their cellular uptake [170,171,172]. When Goodman et al. (2004) compared the toxicity of gold NPs with cationic functional groups (ammonium groups) in comparison to NPs with anionic groups (carboxylate group), their data showed reduced cytotoxicity and cellular uptake with anionic NPs [173]. These studies suggest that changing the surface properties of CNTs and gold nanoparticles with anionic groups could potentially decrease their toxicity. 

The hypothesis that altering the surface properties of NPs can reduce their toxicity is further supported by studies with iron oxide NPs, whose toxicity is attributed to the release of hydroxyl radicals resulting from reactions at their surface [174]. Surface functionalization of iron oxide NPs with organic compounds such as aldehyde, carboxyl and amino groups, stabilized the high chemical activity of these NPs, which resulted in a decrease in toxicity while increasing their biological compatibility [13,175,176,177]. 

**Figure 5:**
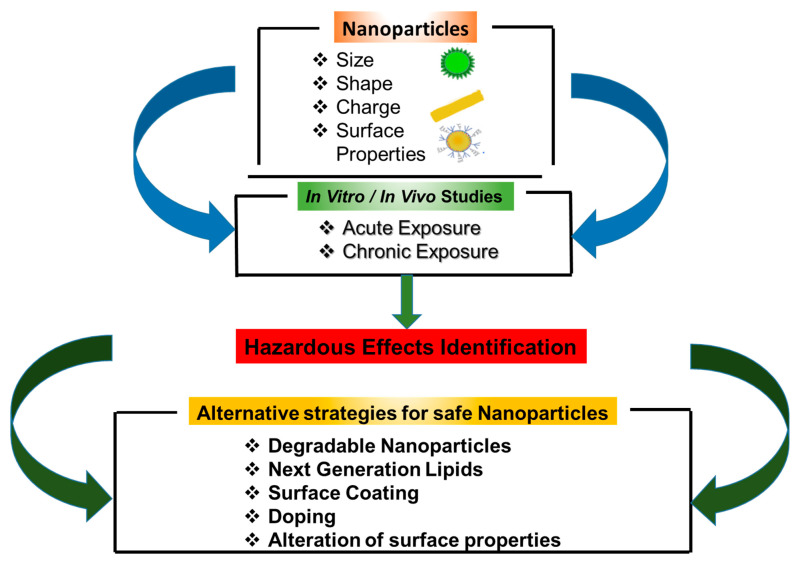
Strategies for safer nanoparticles. Effective strategies start from choosing the right physicochemical properties of NPs. Once studies show potential risks then alternative strategies may include modifications of the composition or surface functionalities in addition to other strategies discussed in the text.

One common theme amongst techniques to reduce or prevent the toxicity of NPs is the modification of physicochemical properties of NPs, such as dissolution and release of toxic metal ions and agglomeration (Figure 5). However, further studies are needed to assess the effectiveness of these strategies under different exposure conditions and environments. This is especially needed due to the increasing number of new NPs being developed and their expanded use in the fields of biomedical application, drug delivery, diagnosis and imaging. There is also a need to develop well-thought-out and standardized procedures for synthesizing NPs that are suited for their different applications. One caveat to this is that many techniques for NP manufacturing and synthesis are based on studies conducted under non-GLP (Good Laboratory Practices)/non-GMP (Good Manufacturing Practices) environments, using small scale batches. As such, large scale-up production of NPs can create unforeseen impurities, necessitating a re-evaluation of safety protocols as well as assessment of finalized products. A further issue complicating the assessment of the toxicity of NPs is their potential toxicity in combination with drugs and other materials, which has received minimal attention. 

### 8.3. Safe Nanoparticles in the Clinic

It is estimated that about 20% of the NPs rejected during clinical trials are because of safety reasons. The approval process of NPs for human consumption requires assessment of NPs’ fate and toxicity. This is usually accomplished through different pre-clinical and clinical phases and approval is granted by specific regulatory agencies such as the Food and Drug Administration (FDA) or European Medicines Agency (EMA). One challenge of this process is that these agencies may not agree in their assessments of both efficacy and safety of NPs. This requires that standards for safety assessment are established [178]. 

Cancer therapy remains the major application of NPs and it is interesting that most of the strategies for producing safer NPs discussed above resulted in NPs that failed in several pre-clinical studies. Further, most of the NPs approved or currently in clinical trials are soft NPs including lipid-based, micelles or polymeric NPs. Among the strategies employed to reduce NP toxicity in the clinic, surface coating using polyethylene glycol (PEG) is the most commonly used method [179]. However, even those NPs approved for clinical use may still develop toxicity. The best example is the liposomal formulation of doxorubicin, Doxil^®^ (Centocor Ortho Biotech, Horsham, PA, USA), which showed efficacy in the treatment of recurrent breast cancer and Kaposi’s sarcoma. However, Doxil^®^ has been associated with the development of skin disorder known as “hand-foot syndrome” and recent studies reported that patients have developed cutaneous squamous cell carcinoma following a repetitive treatment with the pegylated liposomal doxorubicin formulation [179,180]. It is not clear though if these toxicities are related to the drug doxorubicin, to the liposomal carriers, or both. While generally considered non-toxic, the use of PEG has raised concerns. The development of antibodies and the immune response to PEG has been observed clinically, but further research is needed [181]. Nonetheless, these findings highlight the urgent need for studies assessing the long-term effect and toxicity of treatments with NPs. 

It is possible that a complete safety profile of all NPs may never be achieved and that unforeseen potential toxicities may not appear until the NP products are in the market and used in patient therapies. This is true of all drugs and not exclusive to NPs. Enhanced collaboration between academic and basic science researchers and pharmaceutical industries may help facilitate greater advances and facilitate enhanced testing and safety.

## 9. Conclusions

With the application of nanotechnology experiencing log-phase growth, the impact of NPs on cellular and animal models needs to keep pace. Complete toxicological profiling of NPs and development of structure–activity relationships will help identify the key physical or chemical properties of NPs that cause their toxicity and help design safer strategies to minimize NP toxicity by optimizing their physicochemical properties while maximizing their biological efficacies [151,154,182]. Current data on the toxicity of NPs in mammalian cells and tissues suggest that studies are needed to focus on gaining additional insights underlying their toxicity, as well as developing strategies to minimize and prevent the toxicity of NPs. Such strategies need to take in consideration both acute and also chronic exposures to NPs and different exposure routes and environments. Further, dissolution of NPs can have a major effect on their toxicity and soluble NPs appear to be toxic compared to insoluble NPs. Studies have shown that stabilized metal oxide NPs have decreased toxicity compared to non-modified NPs. Future work also should focus on the fate of NPs in biological systems and how organisms react to the long-term exposure of NPs. Finally, it is important to acknowledge the difficulty in identifying effective strategies to produce safer NPs, until a comprehensive understanding of the toxicological status of existing NPs is completed. 

## Abbreviations

NPs	nanoparticles
PNPs	polymeric nanoparticles
PLGA	poly-lactic glycolic acid
FDA	Food and Drug Administration
EMA	European Medicines Agency
MRI	magnetic resonance imaging
CNT	carbon nanotubes
SWCNTs	single-walled carbon nanotubes
MWCNTs	multi-walled carbon nanotubes
EPR	enhanced permeability effect
RES	reticuloendothelial system
PEG	polyethylene glycol
NE	nanoemulsions
ROS	reactive oxygen species
DDT	dichlorodiphenyltrichloroethane
GLP	good laboratory practice
GMP	good manufacturing practice
GRAS	generally recognized as safe
TiO_2_	titanium oxide
FSP	flame spray pyrolysis
ZnO	zinc oxide
